# Pathomechanism of Fever‐Induced Liver Failure in NBAS Deficiency and Treatment Effect of NAC—Observations In Vitro and In Vivo

**DOI:** 10.1111/liv.70762

**Published:** 2026-06-25

**Authors:** Tian Sun, Nicole Hammann, Lina Leghlam, Bianca Peters, Cigdem Arikan, Sarah M. Bedoyan, Qian Chen, Tal Dattner, Felix Distelmaier, Alexander Fichtner, Sven F. Garbade, Nedim Hadžić, Robert Hegarty, Andrea Hellwig, Marianne Hørby Jørgensen, Carolin Jentsch, Sabine Jung‐Klawitter, Martin Laass, Elke Lainka, Hannah Münch, Halise Neslihan Önenli Mungan, Moritz Niesert, Stephanie Oehrl, Begona Polo, Knut Schäkel, James E. Squires, Stefan Kölker, Georg F. Hoffmann, Christian Staufner, Dominic Lenz

**Affiliations:** ^1^ Division of Paediatric Neurology and Metabolic Medicine Heidelberg University Medical Faculty Heidelberg, Centre for Paediatric and Adolescent Medicine Heidelberg Germany; ^2^ Department of Paediatric Gastroenterology and Organ Transplant Koç University School of Medicine Istanbul Turkey; ^3^ Department of Paediatric Gastroenterology and Hepatology UPMC Children's Hospital of Pittsburgh Pittsburgh Pennsylvania USA; ^4^ Department of Dermatology Heidelberg University Heidelberg Germany; ^5^ Department of General Paediatrics, Neonatology and Paediatric Cardiology Heinrich‐Heine‐University, University Children's Hospital Düsseldorf Germany; ^6^ King's College Hospital London UK; ^7^ Paediatric Liver, GI and Nutrition Centre King's College Hospital NHS Foundation Trust London UK; ^8^ Department of Neurobiology, Interdisciplinary Centre for Neurosciences (IZN) Heidelberg University Heidelberg Germany; ^9^ Rigshospitalet, Paediatrics and Adolescent Medicine Copenhagen Denmark; ^10^ Department of Paediatrics Dresden University of Technology Dresden Germany; ^11^ Department for Paediatric Nephrology, Gastroenterology, Endocrinology and Transplant Medicine, Paediatrics II University of Duisburg‐Essen, University Hospital Essen Essen Germany; ^12^ Department of Paediatrics, Division of Paediatric Metabolism Cukurova University Adana Turkey; ^13^ Pediatrica Gastrohepathology Unit Hospital Universitario y Politécnico La Fe, University La Fe Hospital Valencia Spain

**Keywords:** infantile liver failure syndrome‐2 (ILFS2), *N*‐acetylcysteine (NAC), neuroblastoma amplified sequence (NBAS), paediatric acute liver failure (PALF), reactive oxygen species (ROS)

## Abstract

**Background and Aims:**

Pathogenic variants in *neuroblastoma amplified sequence* (*NBAS*) gene causes infantile liver failure type 2 (IFLS2; MIM 616483), characterised by recurrent episodes of liver failure triggered by febrile infections. The underlying pathophysiological mechanisms remain incompletely understood. With this work we try to shed light on the pathomechanism and propose a potential therapeutic option.

**Methods:**

For in vitro analyses, human skin fibroblasts were obtained from three individuals with ILFS2 and one healthy control. Cells were cultivated at 37°C or 40°C. Western blots were performed to assess NBAS protein and its interaction partners. Furthermore, immunofluorescence and electron microscopy were used to examine morphological changes associated with endoplasmic reticulum (ER) stress. Apoptosis was measured using flow cytometry. The effects of *N*‐acetylcysteine (NAC) treatment were analysed not only in cultured fibroblasts but also in vivo retrospectively in 16 affected individuals.

**Results:**

We demonstrate that elevated temperature induces ER stress in fibroblasts from individuals with *NBAS* variants, leading to increased reactive oxygen species (ROS) production and apoptosis. Treatment with the antioxidant NAC, an established therapeutic in acetaminophen‐induced liver failure, effectively mitigated oxidative stress and reduced apoptosis in vitro. In a retrospective clinical analysis, there was a trend that NAC treatment was associated with reduced severity of hepatic crises, suggesting a potential therapeutic benefit.

**Conclusions:**

Our findings link fever‐induced ER stress, ROS accumulation, and apoptosis to the pathogenesis of liver failure in NBAS deficiency. NAC attenuates these cellular stress responses and may represent a promising supportive treatment option in NBAS‐associated liver failure.

## Key Points

- When exposed to elevated temperatures, neuroblastoma amplified sequence (NBAS)‐deficient fibroblasts experience significant endoplasmic reticulum stress, a marked increase in reactive oxygen species (ROS) levels, and an induction of apoptosis.
- *N*‐acetylcysteine (NAC) plays a pivotal role in protecting NBAS mutant fibroblasts from ROS and subsequent apoptosis.
- In individuals with NBAS deficiency, the application of NAC during febrile illness may reduce the severity of hepatic crises.

## Lay Summary

Genetic changes to both alleles of the *NBAS* gene lead to, among others, recurrent episodes of fever‐triggered liver failure. However, the reason why fever causes liver damage has not been understood.

In this study, we examined skin cells from people with NBAS deficiency and compared them with cells from a healthy individual. When the cells were exposed to a higher temperature (similar to what happens during a fever), the NBAS‐deficient cells showed signs of stress within a part of the cell called the endoplasmic reticulum (ER) and there was a higher production of reactive oxygen species (ROS). Furthermore, many of the affected cells underwent programmed cell death.

We then tested the effect of an antioxidant medication (called *N*‐acetylcysteine; NAC) used to treat certain types of liver failure. In laboratory experiments, NAC reduced the build‐up of harmful ROS and protected the NBAS‐deficient cells from dying. Furthermore, we reviewed clinical data from 16 people with NBAS deficiency. NAC treatment during fever‐related illnesses seemed to lessen the severity of liver crises.

Overall, our study suggests that fever can trigger a chain reaction in NBAS deficiency—causing cellular stress, oxidative damage and cell death—which may contribute to liver failure. NAC appears to interrupt this process and might be a useful treatment during febrile illness in people with NBAS deficiency.

**Abbreviations**
Ab(s): antibody(ies)
ALF: acute liver failure
ALT: alanine aminotransferase
AST: aspartate aminotransferase
ATF6: activating transcription factor 6
BiP: binding immunoglobulin protein
BSA: bovine serum albumin
DAPI: 4′,6‐diamidino‐2‐phenylindole
DCFDA: 2′,7′‐dichlorodihydrofluorescein diacetate
DLDD: dihydrolipoamide dehydrogenase deficiency
D‐MEM: Dulbecco's modified eagle's medium
eIF2α: eukaryotic initiation factor 2
ER: endoplasmic reticulum
ERGIC: ER (endoplasmic reticulum)‐Golgi intermediate compartment
FACS: fluorescence‐activated cell sorting
FBS: fetal bovine serum
Fig: figure
FITC: fluorescein isothiocyanate
GM130: golgi matrix protein 130 kDa
GGT: gamma‐glutamyl‐transferase
HSP70: heat shock protein 70
ICU: intensive care unit
ILFS2: infantile liver failure syndrome type 2
IV: intravenous
INR: international normalised ratio
IRE1α: inositol‐requiring enzyme 1α
MASLD: metabolic dysfunction‐associated steatotic liver disease
MIM: Mendelian inheritance in man
NAC: *N*‐acetylcysteine
NBAS: neuroblastoma amplified sequence
NH3: ammonia
NMD: nonsense‐mediated mRNA decay
NRZ: nBAS‐RINT1‐ZW10
PALF: paediatric acute liver failure
PBS: phosphate‐buffered saline
PDI: protein disulfide isomerase
PERK: protein kinase RNA‐like endoplasmic reticulum kinase
PI: propidium iodide
PO: peroral
PVDF: polyvinylidene fluoride
RINT1: Rad50‐interacting protein 1
RIPA: radioimmunoprecipitation assay
ROS: reactive oxygen species
TEM: transmission electron microscopy
UPF1: up‐frameshift protein 1
UPR: unfolded protein response
WES: whole exome sequencing
WGS: whole genome sequencing
WRS: Wolcott–Rallison syndrome
ZW10: zeste white 10

**Units**
°C: degree celsius
g: gram
kDa: kilodalton
kg: kilogram
kV: kilovolt
L: litre
mg: milligram
mL: millilitre
μg: microgram
μL: microlitre

## 1 Introduction

Paediatric acute liver failure (PALF) is a rare but life‐threatening clinical condition characterised by the sudden onset of liver injury and severely impaired liver function in children without known chronic liver disease. It mainly occurs in the first year of life [1]. PALF is caused by infections, metabolic disorders, drugs, toxins, and autoimmune diseases; however, in up to 50% of cases, the cause has remained indeterminate [1, 2].

In recent years, whole exome/genome sequencing (WES/WGS) uncovered novel aetiologies including disorders of vesicular trafficking as a relevant group of PALF. Within this category, infantile liver failure syndrome type 2 (ILFS2; MIM: 616483) due to variants in Neuroblastoma Amplified Sequence (*NBAS*) is the most predominant one [3, 4, 5]. NBAS deficiency is an autosomal recessive disorder affecting various organ systems, including the liver, skeletal system, nervous system (with eye involvement), immune system and muscles [6]. Three distinct clinical subgroups were identified, depending on the affected protein domain/region (C‐terminal region, Sec39 domain, *β*‐propeller domain) [6]: SOPH syndrome, characterised by short stature, optic atrophy, and Pelger–Huët anomaly (MIM: 614800) [7], ILFS2 [5], and a combined phenotype.

As febrile infections have been observed to trigger liver decompensation, affected individuals are administered early antipyretic treatment. Furthermore, an anabolic state is ensured by dextrose and lipid infusions—a concept known from various inherited metabolic diseases [8]. Shedding light on the pathomechanistic background is essential for the identification of further, more specific treatment targets.

Regarding pathophysiology, data are sparse. NBAS is a component of the NBAS‐RINT1‐ZW10 (NRZ) tethering complex, composed of Rad50‐interacting protein 1 (RINT1), Zeste white 10 (ZW10), and p31. Together, they mediate vesicular trafficking between the Golgi apparatus and the endoplasmic reticulum (ER) inside human cells [4, 9, 10]. Previous studies have shown that the transcription of ER stress‐related genes is increased in NBAS‐deficient fibroblasts [5].

In our study, we analysed the ER stress effector pathways, oxidative stress, and apoptosis in NBAS‐deficient fibroblasts. Furthermore, administration of *N*‐acetylcysteine (NAC), an anti‐oxidative drug used in PALF due to acetaminophen intoxication, was evaluated as a therapeutic option in fibroblasts. Additionally, clinical data from individuals with biallelic pathogenic *NBAS* variants treated with or without NAC were analysed.

## 2 Materials and Methods

### 2.1 Cell Culture

All experiments were conducted in vitro using human fibroblasts obtained from individuals diagnosed with biallelic pathogenic variants affecting the Sec39 domain of *NBAS* and presenting an ILFS2 phenotype (Table 1) [6]. Fibroblasts from a healthy donor were used as a control. Fibroblasts were cultivated in Dulbecco's Modified Eagle Medium (D‐MEM) supplemented with 10% fetal bovine serum (FBS), 1% penicillin–streptomycin, 1% non‐essential amino acids, and 0.001% *β*‐mercaptoethanol at 37°C or at 40°C for 48 h for temperature‐dependent experiments.

**TABLE 1:** Genotype of the patients included in this study and the control.

ID	Subgroup	Allele 1	Exon	Allele 2	Exon
NBAS 2	Sec39	c.(2708T > G)	24	c. (2708T > G)	24
NBAS 9	Sec39	c.(2951T > G)	26	c. (2827G > T)	25
NBAS 5	Sec39	c.(3164T > C)	28	c. (3010C > T)	26
N21	Wildtype			Wildtype	

### 2.2 Reactive Oxygen Species (ROS) Detection

Cells were seeded in 6‐well plates or glass‐bottom dishes at 50%–70% confluency and cultured at 37°C with 5% CO_2_ for 24 h prior to treatment. Cells maintained at 37°C for an additional 48 h served as controls. For heat stress experiments, cells were incubated at 40°C for 48 h. For antioxidant treatment, cells were pre‐incubated with 20 mM *N*‐acetylcysteine (NAC) for 30 min, and NAC concentration was maintained throughout the entire 48 h incubation at 40°C. Intracellular reactive oxygen species (ROS) levels were assessed using 2′,7′‐dichlorofluorescein diacetate (DCFDA). A 10 μM DCFDA working solution was prepared in serum‐free, phenol red‐free culture medium. After removal of the culture medium, cells were incubated with DCFDA at 37°C for 30 min in the dark. Cells were then washed twice with phosphate‐buffered saline (PBS) to remove excess probe and immediately imaged. Fluorescence images were acquired using a fluorescence microscope (excitation 488 nm, emission 525 nm) with identical acquisition settings for all conditions. Quantification of fluorescence intensity was performed using ImageJ software (NIH, version 1.25G) with background subtraction. Relative fluorescence intensity was normalised to the 37°C control condition.

### 2.3 Western Blotting

For immunoblotting, cells were collected by trypsinisation, washed with PBS, and lysed in radioimmunoprecipitation assay (RIPA) buffer supplemented with protease inhibitors. Protein concentrations were determined using a BCA assay, and 35 μg of total protein per sample were separated on 8%–12% SDS–polyacrylamide gels and transferred onto polyvinylidene fluoride (PVDF) membranes. Membranes were blocked for 1 h at room temperature in blocking buffer consisting of PBS containing 0.1% Tween‐20 and 5% (w/v) non‐fat dry milk. Primary antibodies were incubated overnight at 4°C in blocking buffer. Membranes were then washed with PBST (PBS supplemented with 0.1% Tween‐20) before incubation with horseradish peroxidase (HRP)‐conjugated secondary antibodies diluted in blocking buffer for 1 h at room temperature. Protein bands were visualized using Pierce ECL Western Blotting Substrate (Thermo Fisher Scientific) and imaged with a Fusion FX imaging system (Vilber Lourmat). Densitometric analyses were performed using ImageJ software (NIH, version 1.25G), and protein expression levels were normalised to vinculin or *β*‐actin as loading controls. The following primary antibodies were used: NBAS (1:1000; Sigma‐Aldrich; #HPA036817), inositol‐requiring enzyme 1α (IRE1α) (1:1000; Cell Signalling Technology; #3294), protein kinase RNA‐like ER kinase (PERK) (1:1000; Cell Signalling Technology; #5683), vinculin (1:1000; Thermo Fisher Scientific; #700062), and *β*‐actin (1:1000; Santa Cruz Biotechnology; #SC‐47778). HRP‐conjugated secondary antibodies were obtained from Sigma‐Aldrich (#A0545) and used at a dilution of 1:1000.

### 2.4 Quantitative Proteomic Analysis

Cells were cultured in T75 flasks to ~90% confluency and, where indicated, incubated at 40°C for 24 h or 48 h prior to harvesting. Cells were washed with PBS, collected by scraping, and pelleted by centrifugation. Cell pellets were lysed in RIPA buffer (Carl Roth GmbH + Co. KG, Karlsruhe, Germany; 23 T1.2), supplemented with protease inhibitor cocktail (Roche, Basel, Switzerland; ref. 04693132001) and incubated on ice for 30 min. Lysates were mechanically disrupted by repeated passage through a fine‐gauge needle and clarified by centrifugation at 13000 rpm for 20 min at 4°C. Protein extracts were subsequently analysed by tandem mass tag (TMT)‐based quantitative proteomics by the proteomics core facility at the European Molecular Biology Laboratory.

### 2.5 Immunofluorescence

Cells were seeded on glass coverslips placed in 12‐well plates at a density of 1 × 10^4^ cells per well and allowed to adhere overnight. After incubation at the indicated temperatures, cells were washed three times with PBS and fixed with 4% formaldehyde in PBS for 15 min at room temperature. Following fixation, cells were washed three times with PBS and permeabilised with 1% Triton X‐100 in Dulbeccos’ phosphate‐buffered saline (DPBS) for 10 min at room temperature. After washing with PBS, non‐specific binding sites were blocked by incubation with 5% bovine serum albumin (BSA) in PBS for 1 h at room temperature. Cells were then incubated with primary antibodies diluted in blocking buffer overnight at 4°C. The following primary antibodies were used: protein disulfide isomerase (PDI) (1:100; Invitrogen; MA3019), Golgi matrix protein 130 kDa (GM130) (1:100; BD Biosciences; 610 823), and ER‐Golgi intermediate compartment protein 53 (ERGIC53) (1:100; Santa Cruz Biotechnology; SC‐365158). After three washes with PBS, cells were incubated with the appropriate Alexa Fluor–conjugated secondary antibodies diluted in blocking buffer for 1 h at room temperature in the dark. The following secondary antibodies were used: Alexa Fluor 488 anti‐rabbit (1:500; Invitrogen; A11034), Alexa Fluor 488 anti‐mouse (1:500; Invitrogen; A10667), Alexa Fluor 555 anti‐rabbit (1:500; Invitrogen; A21428), and Alexa Fluor 555 anti‐mouse (1:500; Invitrogen; A31570). Nuclei were counterstained with 4′,6‐diamidino‐2‐phenylindole (DAPI) (Sigma‐Aldrich; D9542) diluted in PBS. Coverslips were washed with PBS and mounted onto glass slides using Fluoromount‐G (Invitrogen; 00‐4958‐02). Images were acquired using a Leica DM4000 M microscope at 20× and 100× magnifications. Exposure times and detector gains were kept constant across all experimental conditions using LAS X software (Leica) to allow quantitative comparison.

### 2.6 Transmission Electron Microscopy (TEM)

For transmission electron microscopy cells were fixed in 2% glutaraldehyde in 0.1 M sodium phosphate buffer, pH 7.4 and washed. Samples were post‐fixed with 1% osmium tetroxide (OsO4) and 1.5% potassium ferrocyanide, contrasted with uranyl acetate, dehydrated in ethanol and embedded in glycid ether 100‐based resin. Ultrathin sections were prepared using a Reichert Ultracut S ultramicrotome (Leica Microsystems), contrasted and examined with a Zeiss EM 10 CR transmission electron microscope at an acceleration voltage of 60 kV.

### 2.7 Flow Cytometry Detection of Apoptosis Using Annexin V/Propidium Iodide

Apoptosis and necrosis were quantified by flow cytometry using Annexin V–FITC/propidium iodide (PI) staining. Fibroblasts cultured in T25 flasks were washed twice with PBS to remove residual medium and serum, then detached using trypsin–EDTA solution. Proteolysis was stopped by adding an equal volume of growth medium containing serum. Cells were collected by centrifugation (800–1000× *g* for 5 min at 4°C), and the supernatant was discarded. The cell pellet was resuspended in Annexin V binding buffer at a concentration of approximately 1–5 × 10^6^ cells/mL. For staining, 100 μL of cell suspension (corresponding to 1–5 × 10^5^ cells) were transferred into flow cytometry tubes. Cells were stained with 5 μL Annexin V‐FITC and 5 μL propidium iodide (Annexin V‐FITC/PI Apoptosis Detection Kit, Carl ROTH; 7735.1), gently mixed, and incubated. After incubation, 400 μL of 1× Annexin V binding buffer was added to each tube to obtain a final volume of 500 μL. Also, single‐colour‐compensation probes were prepared. Data acquisition was performed using a Gallios Flow Cytometer (Beckman Coulter), and data were analysed using Kaluza software (Beckman Coulter).

### 2.8 Evaluation of a Treatment With NAC in Affected Individuals

Individuals with biallelic pathogenic *NBAS* variants and frequent and/or severe hepatic crises were treated with NAC as part of an individualised emergency protocol (see Infobox 1; currently used protocol). In all individuals, protocols included early continuous antipyretic treatment and prevention of catabolism during infections. At first signs of hepatic involvement, oral NAC was administered at home (150 mg/kg/d in 3 doses) and the patient was referred to the emergency department for laboratory tests. In case of hepatic involvement, intravenous (IV) dextrose (for age dependent dosage see Infobox 1), IV lipids (1 g/kg/d), and IV or peroral (PO) NAC (in acetaminophen intoxication dosages) were administered. These episodes were then compared with previous episodes without administration of NAC as part of their individualised emergency protocol. All procedures were performed in accordance with the ethical standards of the responsible committee on human experimentation (institutional and national) and with the Helsinki Declaration of 1975, as revised in 2024. Informed consent was obtained from all patients, or from their parents in case of minor patients, except for cases where patient data were retrieved from publications. The study was approved by the ethical committee of the Medical Faculty Heidelberg (study number: S‐035_2014; S‐456_2019).

Data on crises with and without NAC administration were collected using case report forms. For each crisis, detailed information was obtained, including basic data (age, length of hospital stay, admission to the intensive care unit (ICU), death due to crisis), clinical symptoms (fever, vomiting, drowsiness, grade of hepatic encephalopathy and others), type of infection and laboratory values aspartate aminotransferase (AST), alanine aminotransferase (ALT), gamma‐glutamyl‐transferase (GGT) and international normalised ratio (INR), on days 1, 2, 3, 5 and 7, as well as the maximal values. Therapeutic management (antipyretics, dextrose, lipids, vitamin K and others) and the type of NAC protocol, including duration and delay after onset of symptoms, were obtained when available.

Infobox 1 **Example of our currently locally used emergency protocol including *N*‐acetylcysteine (NAC) for NBAS deficiency.**

In case of fever > 38°C or recurrent vomiting or drowsiness:

Antipyretics (metamizole, if necessary, acetaminophen, ibuprofen).

Glucose i.v. (g/kg/d) + insulin (if necessary).

**Table:** 

0–12 months	1–3 years	4–10 years	11–15 years	> 16 years
12–15	10–12	7–10	4–7	3–5

Lipid infusion i.v. 1–2 g/kg/d.

NAC 3 × 50 mg/kg p.o.; consider first dose at home.

In case of liver failure: NAC according to acetaminophen intoxication protocol:

- Oral: initially 140 mg/kg, then 70 mg/kg every 4 h for 72 h.
- Intravenous: loading those 150 mg/kg over 60 min, then 50 mg/kg over 4 h, then 100 mg /kg over 16 h (total: 300 mg/kg over 21 h).

### 2.9 Statistical Analysis

Data of in vitro tests are presented as the mean ± SEM of at least three independent biological replicates. Clinical data are presented as mean ± SD, median with minimum and maximum values, and absolute numbers with percentages as appropriate.

Comparisons of in vitro results between the two groups were conducted using unpaired two‐tailed Student's *t*‐test, as indicated in the figure legends. Proteomics data were processed using FragPipe and analysed in the R statistical environment. Contaminants and reverse protein identifications were removed prior to analysis, and proteins quantified with at least two razor peptides were retained. Data were log2 transformed, batch corrected, normalised, and analysed using the limma package. Differentially abundant proteins were identified using a false discovery rate (FDR) threshold of < 0.05 and an absolute fold change > 1.5.

To determine the in vivo differences between the groups (episodes treated with versus without NAC) generalised linear mixed models with a Gamma family and a logarithm link function were performed. As all laboratory tests were performed in fibroblasts from individuals with ILFS2 phenotype, this was included as a covariate alongside age. A *p* < 0.05 was considered as a statistically significant difference. All statistical analyses were performed using R, Microsoft Excel, or GraphPad Prism version 9.0 (GraphPad Software Inc.). Tables were created using Microsoft Office (manufacturer); all graphs were designed using GraphPad Prism version 10.0 (GraphPad Software Inc.).

## 3 Results

### 3.1 Temperature‐Dependent Expression of NBAS and Associated Proteins

**FIGURE 1:**
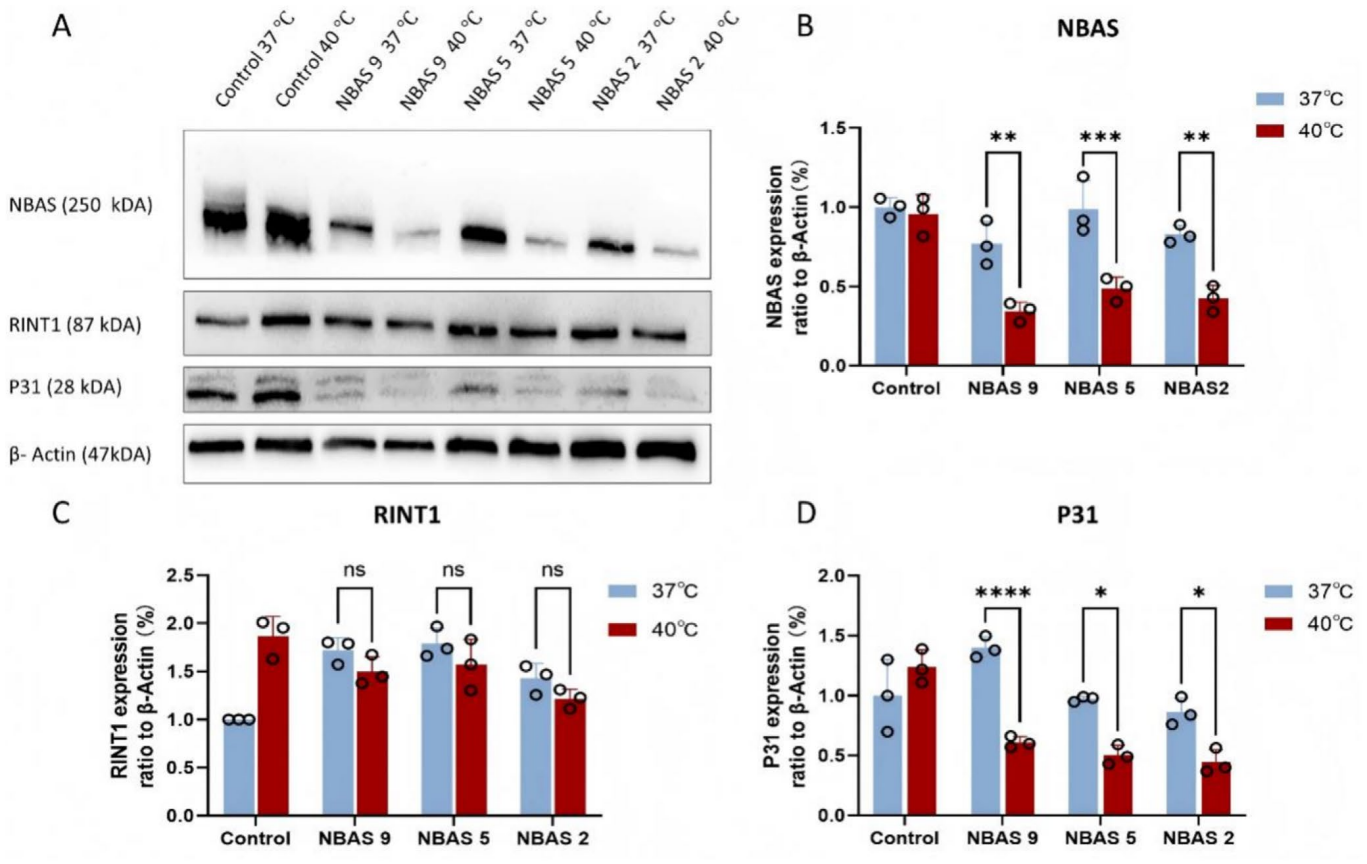
Temperature‐dependent expression of NBAS and associated vesicular transport proteins. (A) Western blot analysis of NBAS, RINT1, and p31 in control and NBAS‐deficient fibroblasts following 48 h incubation at 37°C or 40°C. *β*‐Actin served as a loading control. (B–D) Densitometric quantification of NBAS (B), RINT1 (C), and p31 (D) protein levels normalised to *β*‐Actin. Data are presented as mean ± SEM from three independent experiments (*n* = 3). Statistical significance: *p < 0.05, **p < 0.01, ***p < 0.001, ****p < 0.0001; n.s., not significant.

Western blotting demonstrated a reduction of NBAS protein in NBAS‐deficient fibroblasts at 40°C compared with the physiological temperature of 37°C. A parallel decrease in the expression of the vesicular transport protein p31 was observed, whereas RINT1 expression remained largely unchanged (Figure 1).

### 3.2 Heat‐Induced Disruption of ER–Golgi Organisation and Activation of ER Stress Signalling

We next examined whether the heat‐induced decrease in NBAS and p31 expression was accompanied by structural alterations of the ER‐ and Golgi organisation.

**FIGURE 2:**
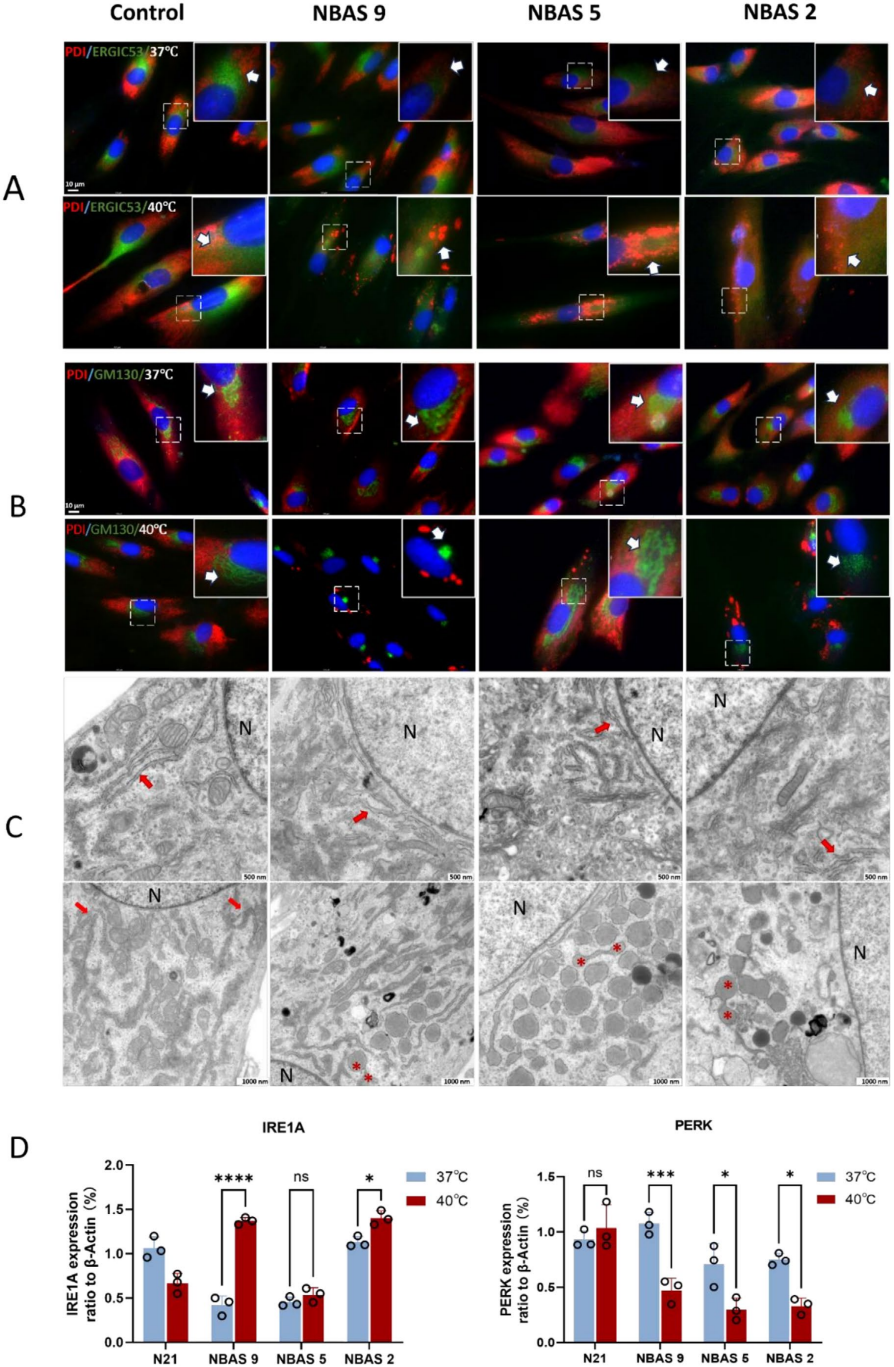
Heat‐induced disruption of ER–Golgi organisation and activation of ER stress signalling. (A) Immunofluorescence staining of PDI (red) and ERGIC53 (green) after 48 h incubation at 37°C or 40°C. Insets show magnified regions. Arrows indicate condensation of the reticular PDI‐positive ER network into aggregates at 40°C in NBAS‐deficient fibroblasts. (B) Immunofluorescence staining of PDI (red) and GM130 (green). Arrows indicate the Golgi apparatus, which gets fragmented and forms compact perinuclear GM130‐positive aggregates in NBAS‐deficient fibroblasts following heat exposure. Nuclei were counterstained with DAPI (blue). Scale bars: 10 μm. (C) Transmission electron microscopy (TEM) images showing ER morphology. Arrows mark ER cisternae and stars mark ER dilatations. Scale bars: 500 nm (upper row) and 1000 nm (lower row). (D) Western blot analyses of IRE1α and PERK expression after incubation at 37°C or 40°C with corresponding densitometric quantification. Data are presented as mean ± SEM from three independent experiments (*n* = 3). *p < 0.05; **p < 0.01; ***p < 0.001; ****p < 0.0001; n.s., not significant.

Heat exposure (40°C) resulted in structural disruption of ER and Golgi compartments in NBAS‐deficient fibroblasts. At 37°C, PDI displayed a reticular ER distribution, whereas incubation at 40°C led to its reorganisation into condensed patches (Figure 2A, arrows; inset). ERGIC53 exhibited a more dispersed pattern following heat exposure, and GM130 often lost its characteristic ribbon‐like morphology, forming compact perinuclear aggregates at 40°C (Figure 2B, arrows; inset), consistent with disruption of the ER–Golgi organisation.

Transmission electron microscopy (TEM) confirmed these alterations. Control cells exhibited ER cisternae, whereas heat‐treated NBAS‐deficient fibroblasts frequently displayed ER (lumen) dilatations (Figure 2C, stars).

In parallel with these structural changes, IRE1α protein levels increased at 40°C, whereas PERK expression was reduced (Figure 2D), indicating activation of ER stress‐associated signaling pathways. Levels comparisons in TMT‐based proteomic analyses showed increased ATF6 expression in controls and NBAS‐deficient fibroblasts at 40°C compared with 37°C with no significant group differences (Figure S4; Table S5). A cleaved form of ATF6 was not detected.

### 3.3 Heat Stress Elevates ROS Levels, Which Are Attenuated by NAC

**FIGURE 3:**
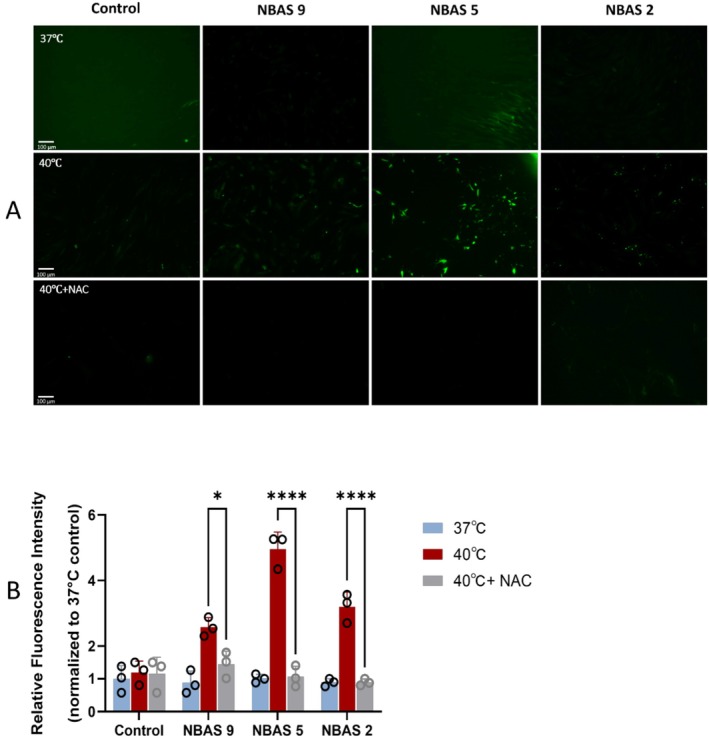
Heat‐induced ROS production and its attenuation by NAC. (A) Representative fluorescence images of DCFDA staining in control and NBAS‐deficient fibroblasts after incubation at 37°C, 40°C, or 40°C with *N*‐acetylcysteine (NAC, 20 mM) administered throughout the 48 h incubation period at 40°C. Scale bars: 100 μm. (B) Quantification of relative fluorescence intensity normalised to the respective 37°C condition. Data are presented as mean ± SD from three independent experiments (*n* = 3). *p < 0.05, **p < 0.01, ***p < 0.001, ****p < 0.0001 vs. 37°C; n.s., not significant.

We next examined whether heat‐induced ER stress was associated with alterations in intracellular reactive oxygen species (ROS) levels. Cellular ROS levels were quantified using 2′,7′‐dichlorodihydrofluorescein diacetate (DCFDA) staining. Control fibroblasts (N21) maintained stable ROS levels at both 37°C and 40°C. In contrast, NBAS2 and NBAS5 fibroblasts exhibited a pronounced increase in fluorescence intensity following incubation at 40°C. Treatment with *N*‐acetylcysteine (NAC, 20 mM) during the entire 48 h incubation period at 40°C significantly attenuated heat‐induced ROS accumulation in NBAS‐deficient fibroblasts (Figure 3).

### 3.4 Heat Stress Induces Apoptosis, Which Is Attenuated by NAC

**FIGURE 4:**
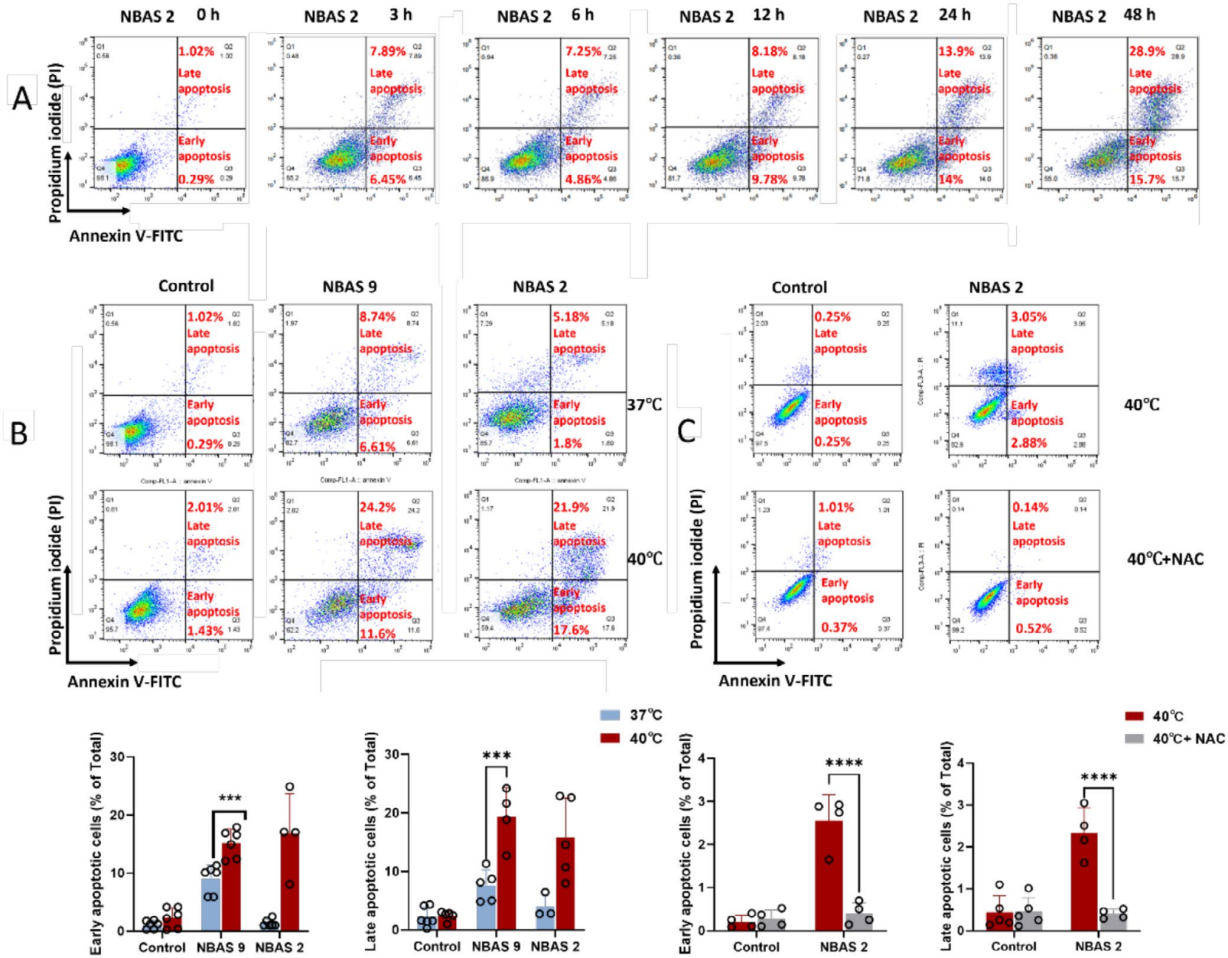
NAC attenuates heat‐induced apoptosis in NBAS‐deficient fibroblasts. (A–C) Flow cytometric analysis of Annexin V/propidium iodide (PI) staining in NBAS‐deficient fibroblasts under heat stress. (A) NBAS2 fibroblasts were exposed to 40°C for 0, 3, 6, 12, 24, and 48 h to assess the time‐dependent induction of apoptosis. (B) Apoptosis levels were compared between NBAS2 and NBAS9 fibroblasts cultured at 37°C or 40°C for 48 h. (C) NBAS2 fibroblasts were incubated at 40°C for 48 h with or without *N*‐acetylcysteine (NAC, 20 mM) to evaluate its cytoprotective effect. The percentage of early and late apoptotic cells was quantified and expressed as mean ± SD (*n* = 3). *p < 0.05, **p < 0.01, ***p < 0.001, ****p < 0.0001 vs. 37°C; n.s., not significant (*p* > 0.05). FITC, fluorescein isothiocyanate.

Apoptosis was evaluated by Annexin V/PI flow cytometry in NBAS‐deficient and control fibroblasts under different temperature conditions (Figure 4A–C). At 40°C, time‐course analysis of NBAS2 fibroblasts revealed a gradual and time‐dependent increase in both early and late apoptotic populations from 3 to 48 h compared with baseline (0 h), indicating progressive induction of apoptosis upon prolonged heat exposure (Figure 4A). Exposure of NBAS‐deficient fibroblasts to 40°C for 48 h resulted in a marked increase in both early and late apoptotic populations compared with cells maintained at 37°C (*p* < 0.01). Quantitative analysis showed significantly higher apoptosis rates in NBAS2, NBAS5, and NBAS9 fibroblasts than in N21 control cells at 40°C (*p* < 0.001, *p* < 0.01, respectively; Figure 4B). In contrast, treatment with NAC (20 mM) markedly reduced both early and late apoptosis in NBAS2 fibroblasts under heat stress (*p* < 0.0001), indicating that oxidative stress contributes to the temperature‐dependent cell death phenotype and that NAC exerts a cytoprotective effect (Figure 4C).

### 3.5 NAC Therapy in Individuals With Biallelic NBAS Variants

We retrospectively analysed 86 hepatic crises in 16 individuals with biallelic pathogenic *NBAS* variants. The affected region/domain of the NBAS protein was classified according to Staufner et al. [6]. Seven individuals had biallelic pathogenic variants affecting the Sec39 domain, two individuals each had variants affecting the *β*‐propeller domain and the C‐terminal region, and the remaining five individuals either had variants affecting interdomain regions or two missense variants affecting different domains.

A total of 49 crises in 12 individuals were treated with NAC, while 37 crises in 12 individuals were managed without NAC. Four individuals experienced only crises without NAC treatment. For another four individuals, data were available exclusively for NAC‐treated episodes. For details see Table S1. Baseline characteristics (pathogen, additional emergency measures) were comparable between episodes treated with NAC and those without. However, as protocols were adapted to include NAC in the disease course, the individuals were older in crises treated with NAC. Furthermore, no side‐effects were reported and administration was overall well tolerated. For an overview of all characteristics of crises treated with versus without NAC in the overall cohort and in individuals with ILFS2 see Table S2.

Overproportioned many episodes fulfilling the acute liver failure (ALF) criteria were in individuals from the ILFS2 subgroup treated without NAC, and overproportioned many episodes not fulfilling the ALF criteria were in individuals with NBAS deficiency other than ILFS2 treated with NAC. The maximal INR was lower in patients treated with NAC in the overall and in the ILFS2 group (mean and median). However, mixed linear models showed a significant decrease with age and a significant increase for ILFS2; treatment with NAC had no significant effects. Similarly, AST and ALT in the overall cohort were lower in episodes treated with NAC (mean and median); in the ILFS2 subgroup both were higher in episodes treated with NAC. ALT was significantly lower with increasing age and significantly higher in the ILFS2 group; there were no significant effects of treatment with NAC on either of them. Length of hospital admission was significantly correlated with younger age, and individuals treated with NAC were in tendency admitted longer. Rates of intensive care unit (ICU) admission were not significantly altered by age; they were in tendency higher in patients treated with NAC. Mortality was comparable in the overall cohort, with one death in each group.

Analysis of laboratory parameters over time also revealed no significant differences. However, on day 1, AST, ALT, and INR tended to be higher in the non‐NAC group and subsequently decreased in both groups (Figure S1).

A pathogen‐specific analysis showed no differences between groups. Suspected bacterial infections accounted for 18 crises, and viral infections for 44 crises. Half of the crises triggered by bacterial infections, and approximately 40% of crises triggered by viruses fulfilled ALF criteria. The most frequently identified pathogen was influenza virus (8 episodes), followed by severe acute respiratory syndrome coronavirus 2 (5 episodes), entero−/rhinovirus (3 episodes), rotavirus (2 episodes), and *Moraxella catarrhalis* (2 episodes). Single cases were observed for norovirus, adenovirus, parainfluenza virus, respiratory syncytial virus, *Streptococcus pyogenes* , *Clostridium difficile* , Mycoplasma species, *Escherichia coli* , and *Listeria monocytogenes* . Crises triggered by influenza viruses were associated with a significantly higher incidence of ALF compared with other viral infections (75% in both groups; *p* = 0.03; Table S3 and Figure S2).

Finally, the analysis of crises at an individual‐level in patients who experienced both NAC‐treated and untreated crises (NBAS53, NBAS84, NBAS143, NBAS147, NBAS148) showed that the frequency of ALF was lower in three and the same in two patients upon treatment with NAC (Table S4). Mean INR, AST, and ALT were higher in two and lower in three patients upon treatment with NAC; interestingly, in all individuals, the highest transaminase values were observed in crises not treated with NAC (see Table S4 and Figure S3).

## 4 Discussion

### 4.1 Pathophysiology of ALF in NBAS Deficiency

Current knowledge about the pathophysiology of NBAS deficiency is limited. Staufner et al. demonstrated that NBAS protein expression decreases upon elevated temperature, accompanied by increased mRNA levels of ER stress markers in vitro [5, 8]. In the present study, we confirmed the upregulation of ER stress markers at the protein level under elevated temperature conditions and further demonstrated increased ROS production and apoptosis in ILFS2.

The ER plays a central role in protein synthesis, folding, and transport, while the Golgi apparatus is responsible for post‐translational modification and sorting. NBAS protein is essential for anterograde and retrograde intracellular trafficking between the Golgi apparatus and the ER [5, 8, 9] and also participates in nonsense‐mediated mRNA decay (NMD), ensuring high‐fidelity mRNA translation [11]. NBAS and the core NMD factor UPF1 (Up‐frameshift protein 1) co‐regulate the stability of ER‐associated transcripts, particularly those involved in the cellular stress response [11].

Accumulation of unfolded or misfolded proteins induces ER stress, activating the unfolded protein response (UPR). During this process, the ER‐based heat shock protein BiP (binding immunoglobulin protein), a member of the HSP70 (heat shock protein 70) protein family, dissociates from the three major ER stress sensors—IRE1α, PERK and ATF6 (activating transcription factor 6)—resulting in their activation [12]. In our study, IRE1α expression was increased, while PERK expression was reduced, suggesting differential activation of UPR signalling pathways. The third canonical sensor in the UPR, ATF6, showed no significant changes. Activation of IRE1α promotes protein refolding, secretion, and degradation of misfolded proteins, as well as the upregulation of genes encoding cytoprotective factors [12]. However, persistent or severe ER stress can shift IRE1α signalling towards pro‐apoptotic pathways [12, 13].

PERK activation reduces global protein synthesis while selectively allowing translation of proteins involved in ER stress resolution, induces cell cycle arrest, and activates antioxidative responses [12, 13]. Chronic PERK signalling, however, can also lead to apoptosis. Sustained or severe ER stress ultimately results in a terminal UPR, characterised by increased ROS production and apoptosis [12]. Reduction of PERK in NBAS deficiency under elevated temperatures is a surprising finding, potentially corresponding to an intact vesicular trafficking being essential for UPR capacity and stress tolerance.

The liver is highly metabolically active and responsible for the synthesis of numerous proteins, making it particularly sensitive to disturbances in UPR signalling [13, 14, 15]. In metabolic dysfunction‐associated steatotic liver disease (MASLD), UPR activation contributes to inflammation and hepatocellular apoptosis, promoting disease progression [14]. Wolcott–Rallison syndrome (WRS), caused by pathogenic variants in *EIF2AK3* encoding the PERK protein, manifests with neonatal diabetes mellitus, multiple epiphyseal dysplasia, and infection‐associated ALF in children [16]. Although the mechanism of ALF in WRS is not fully understood, increased ER stress due to PERK dysfunction and subsequent hepatocellular apoptosis is suspected [17]. Reduced eIF2*α* (eukaryotic initiation factor 2*α*) phosphorylation due to PERK deficiency may impair oxidative stress response, leading to ROS accumulation in WRS [18].

Increased ROS generation is also implicated in ALF caused by other genetic disorders: RINT1 deficiency, affecting a direct interactor of NBAS, leads to elevated ROS production [19], and in dihydrolipoamide dehydrogenase deficiency (DLDD), mitochondrial dysfunction similarly results in increased ROS generation [20]. Taken together, ROS overproduction and activation of integrated stress responses appear to represent common effector mechanisms in monogenic forms of ALF [18].

Our study is the first linking decreased NBAS protein expression with increased ER stress—characterised by elevated IRE1α and reduced PERK levels—alongside enhanced ROS generation and apoptosis under febrile conditions. These findings provide novel insights into the pathomechanism of fever‐induced ALF in NBAS deficiency (ILFS2 subgroup). Future studies will unveil whether the other subgroups share this pathomechanistic pathway.

Importantly, the focus of this work lies on the heat‐induced changes of the cells—the ER particularly; examining the correlate of fever‐induced ALF. However, in NBAS deficiency, abnormalities of the immune system are also frequent [21] and in addition, inflammatory abnormalities such as cytokine dysregulation or interferon activation might add to the pathomechanism and should be explored in the future.

Interestingly, influenza infection led significantly more frequently to ALF in compared with other infectious diseases. This might be due to the fact that influenza A infection itself is causing ROS production (at least in respiratory cells) [22, 23]. In fact, oxidant‐antioxidant imbalances are considered one of the key factors leading to exacerbation of respiratory symptoms [22]. In influenza A infection, part of the pathomechanism is linked to IRE1 activation and ER‐stress, with increased ROS production with effects both on lung tissue and influenza virus [24]. To our knowledge, there are no data linking influenza, ROS and ALF. Nevertheless, it seems likely that additional ER‐stress, ROS or oxidant‐antioxidant imbalances contribute to the development of ALF in individuals with pre‐existing conditions leading to increased ROS generation (such as NBAS deficiency). Similarly, severe crises caused by influenza infection are known in DLDD, which is also characterised by increased ROS production and ALF [20]. Therefore, seasonal influenza vaccination should be strongly recommended for all individuals with NBAS deficiency.

### 4.2 Treatment With NAC In Vitro and In Vivo in NBAS Deficiency

Given the identified pathomechanism involving increased ROS generation, we investigated the potential effects of NAC treatment both in vitro and in vivo. NAC is an established treatment for acetaminophen‐induced liver failure [25]. In non‐acetaminophen‐induced ALF, a large systematic review in adults demonstrated improved transplant‐free survival and shorter hospital stays with NAC treatment [26]. In contrast, a paediatric study reported no overall survival benefit and lower 1‐year transplant‐free survival [27].

Nevertheless, NAC has been investigated in specific genetic disorders where ALF is associated with increased ROS production. Case reports suggest beneficial effects of NAC in WRS [17, 18], and potential efficacy has also been proposed for DLDD, in which mitochondrial dysfunction leads to excessive ROS generation [20]. In transaldolase (TALDO1) deficiency, oxidative stress is thought to play an important role in the development of ALF, caused by NADPH and secondary glutathione depletion. In mice, this could be blocked by treatment with NAC [28]. While there is some clinical overlap between TALDO1 and NBAS deficiency (especially recurrent ALF), other symptoms such as progressive liver fibrosis/cirrhosis, anaemia, pancytopenia, renal manifestations, and cardiac manifestations are rarely observed in NBAS deficiency. Interestingly, a heterozygous *TALDO1* variant is considered a risk factor for acetaminophen‐induced liver failure [29], while this has not been observed in NBAS deficiency. Also, in TRMU deficiency (transient, infantile liver failure), cysteine supplementation in the first year of life improves survival. NAC was suggested as a primary cysteine source because of its additional potential benefits regarding oxidative stress [30].

In our study, NAC administration attenuated ROS generation and apoptosis in fibroblasts. However, no significant differences in crisis severity were observed between NAC‐treated and untreated episodes in affected individuals. This may be due to the small sample size, interindividual variability in treatment response, or delayed initiation of therapy. Additionally, treatment was sometimes started during severe crises in an attempt to achieve a therapeutic effect, potentially confounding the results. Unfortunately, as the group was heterogeneous and the cohort was small, we were not able to perform a time‐to‐treatment stratification. Also, as treatment with NAC was added in the course, the effects of older age and treatment with NAC are difficult to disentangle. Hepatic crises in older children tend to be milder in general; however, they can still be fatal [31]. A trend towards reduced hepatocellular injury and milder impairment of liver function was observed in the NAC group. To disentangle confounders, future prospective studies are needed. As in vitro elevated temperature leads to ER stress and ROS, treatment in vivo would need to be initiated timely with the beginning of the febrile episode in order to effectively prevent ALF. As this is the first study to evaluate NAC therapy in hepatic crises due to NBAS deficiency, definitive conclusions cannot be drawn yet. We hypothesise that a subset of individuals with ALF, in whom ROS generation represents a predominant pathogenic mechanism, may benefit from NAC treatment. Prospective studies are needed to validate this hypothesis and to determine optimal timing and dosage. For future studies, also measurement of glutathione and ophthalmic acid levels as biomarkers of ROS [32] before and after administration of NAC in vivo and in vitro might enhance our understanding of the mechanism of action of NAC treatment in NBAS deficiency. In addition, measuring ROS in other genetic disorders associated with acute liver failure might be helpful to identify patients who will benefit from treatment with NAC in those life‐threatening situations.

## Author Contributions

Tian Sun, Bianca Peters and Dominic Lenz designed the study. Tian Sun and Lina Leghlam conducted the laboratory experiments. Nicole Hammann and Bianca Peters led clinical data collection. Stephanie Oehrl and Qian Chen led FACS data analysis. Arikan Cigdem, Sarah M. Bedoyan, Tal Dattner, Felix Distelmaier, Nedim Hadžić, Robert Hegarty, Andrea Hellwig, Marianne Hørby Jørgensen, Sabine Jung‐Klawitter, Martin Laas, Elke Lainka, Halise Neslihan Önenli Mungan, Hannah Münch, Begona Polo, Knut Schäkel, James Squires, Georg F. Hoffmann, and Christian Staufner contributed to data collection. Dominic Lenz supervised all experiments and clinical data acquisition. Sven Garbade performed statistical analyses. Tian Sun, Lina Leghlam, Bianca Peters and Nicole Hammann together with Dominic Lenz wrote the manuscript, which was critically revised by all authors. All authors approved the final manuscript.

## Funding

This study was supported by the Dietmar Hopp Foundation, St. Leon‐Rot, Germany (grant number: 23011235) and by the German Research Foundation (DFG, project number: 518295121).

## Ethics Statement

The study was approved by the ethical committee of the Medical Faculty Heidelberg (study number: S‐035_2014; S‐4562019).

## Consent

Informed consent was obtained from all patients or their parents or caregivers in case of minor patients, except for cases where patient data were retrieved from publications.

## Conflicts of Interest

The authors declare no conflicts of interest.

## Supporting information


**Table S1:** All individuals with NBAS deficiency included in the clinical evaluation of hepatic crises upon treatment including *N*‐acetylcysteine (NAC) compared with treatment without NAC. For each individual PID, genetic variants including affected region or domain and number of episodes each with and without treatment with NAC.
**Table S2:** Hepatic crises in individuals with NBAS deficiency (all individuals and only individuals with ILFS2 syndrome) upon emergency treatment with N‐acetylcysteine (NAC) compared to emergency treatment without NAC. Frequencies with percentages or mean values with variance and median with range are given.
**Table S3:** Triggering pathogens for hepatic crises in NBAS deficiency including frequency of ALF in dependency of the pathogen and treatment with N‐acetylcysteine. For comparing group differences Chi2‐tests were performed and p < 0.05 are considered significant and marked with a star.
**Table S4:** Comparison of hepatic crises in single individuals with NBAS deficiency treated with and without N‐acetylcysteine (NAC). Mean values with standard deviation or frequencies with percentages are given as appropriate.
**Table S5:** Differential cyclic AMP‐dependent transcription factor ATF‐6 alpha protein abundance analysis of NBAS‐deficient and control fibroblasts from proteomic analysis. Proteins were quantified by TMT‐based proteomics and analysed using the limma framework. Log2 fold changes are shown relative to control samples at the corresponding temperature. Adjusted p values (FDR) were calculated using multiple testing correction. Proteins were considered significant with FDR < 0.05 and absolute fold change > 1.5.
**Figure S1:** The development of the laboratory values INR, AST and ALT is shown for crises treated with NAC (at least one day prior treatment before determination of the laboratory value) compared with crises which were not treated with NAC at least one day prior to taking the blood sample.
**Figure S2:** Frequency of suspected or proven pathogens in all hepatic crises in NBAS deficiency (a) compared with frequencies of pathogens in only crises fulfilling the ALF criteria (b).
**Figure S3:** The laboratory values INR, AST, and ALT for all five individuals with NBAS deficiency and at least 2 crises each upon treatment with and without NAC are shown together with the mean values.
**Figure S4:** ATF6 protein levels in NBAS‐deficient fibroblasts under basal and fever‐mimicking conditions. Relative abundance of ATF6 was quantified by TMT‐based proteomics in control and NBAS‐deficient fibroblast cell lines (Sec39: 2F, 5F) cultured at 37°C and 40°C. Protein levels at 37°C were compared with control cells at 37°C, and levels at 40°C were compared with control cells at 40°C. Data are presented as box‐and‐whisker plots showing three biological replicates per condition. Statistical analysis was performed using the limma framework with multiple testing correction. No significant differences were observed (FDR < 0.05 and absolute fold change > 1.5).

## Data Availability

The data that support the findings of this study are available from the corresponding author upon reasonable request.
