# Supplementary material for: Pathomechanism of Fever‐Induced Liver Failure in NBAS Deficiency and Treatment Effect of NAC—Observations In Vitro and In Vivo
Source: Liver Int. 2026 Jun 25;46(8):e70762. doi: 10.1111/liv.70762 (PMC13305340; doi:10.1111/liv.70762)
Supplement: Supplementary file 1 — Table S1: All individuals with NBAS deficiency included in the clinical evaluation of hepatic crises upon treatment including N‐acetylcysteine (NAC) compared with treatment without NAC. For each individual PID, genetic variants including affected region or domain and number of episodes each with and without treatment with NAC. Table S2: Hepatic crises in individuals with NBAS deficiency (all individuals and only individuals with ILFS2 syndrome) upon emergency treatment with N‐acetylcysteine (NAC) compared to emergency treatment without NAC. Frequencies with percentages or mean values with variance and median with range are given. Table S3: Triggering pathogens for hepatic crises in NBAS deficiency including frequency of ALF in dependency of the pathogen and treatment with N‐acetylcysteine. For comparing group differences Chi2‐tests were performed and p < 0.05 are considered significant and marked with a star. Table S4: Comparison of hepatic crises in single individuals with NBAS deficiency treated with and without N‐acetylcysteine (NAC). Mean values with standard deviation or frequencies with percentages are given as appropriate. Table S5: Differential cyclic AMP‐dependent transcription factor ATF‐6 alpha protein abundance analysis of NBAS‐deficient and control fibroblasts from proteomic analysis. Proteins were quantified by TMT‐based proteomics and analysed using the limma framework. Log2 fold changes are shown relative to control samples at the corresponding temperature. Adjusted p values (FDR) were calculated using multiple testing correction. Proteins were considered significant with FDR < 0.05 and absolute fold change > 1.5. Figure S1: The development of the laboratory values INR, AST and ALT is shown for crises treated with NAC (at least one day prior treatment before determination of the laboratory value) compared with crises which were not treated with NAC at least one day prior to taking the blood sample. Figure S2: Frequency of suspected or proven pathogen [file LIV-46-0-s001.docx]

| **PID** | **Genetic variant Allele 1** | **Affected region/ domain** | **Genetic variant Allele 2** | **Affected region/ domain** | **Episodes with NAC (n)** | **Episodes w/o NAC (n)** |
| --- | --- | --- | --- | --- | --- | --- |
| **NBAS 18** | c.[2330C>A]  p.[Pro777His] | Sec39 | c.[1187G>A],  p.[Trp396*] | ß-propeller | 2 | 2 |
| **NBAS 53** | c.[3602A>C]  p.[Gln1201Pro] | Sec39 | c.[3602A>C]  p.[Gln1201Pro] | Sec39 | 2 | 3 |
| **NBAS 55** | c.[2330C>A]  p.[Pro777His] | Sec39 | c.[2330C>A]  p.[Pro777His] | Sec39 | - | 1 |
| **NBAS 56** | c.[2330C>A]  p.[Pro777His] | Sec39 | c.[2330C>A]  p.[Pro777His] | Sec39 | - | 1 |
| **NBAS 84** | c.[3363A>G]  p.[Ile1121Met] | Sec39 | c.[513+2T>C]  p.[?] |  | 6 | 11 |
| **NBAS 130** | c.[1556T>A]  p.[Val519Glu] | between ß-Propeller and Sec39 | c.[1556T>A]  p.[Val519Glu] | between ß-Propeller and Sec39 | 4 | - |
| **NBAS 131** | c.[307G>A]  p.[Ala103Thr] | ß-propeller | c.[5119T>A]  p.[Phe1707Ile] | C-terminal | 1 | - |
| **NBAS 132** | c.[1226C>T]  p.[Ala409Val] | ß-propeller | c.[1628_1629InsA]  p.[Ser544Valfs*11] |  | 4 | - |
| **NBAS 133** | c.[2330C>A]  p.[Pro777His] | Sec39 | c.[1187G>A]  p.[Trp396*] | ß-propeller | 1 | 3 |
| **NBAS 135** | c.[3602A>C]  p.[Gln1201Pro] | Sec39 | c.[3602A>C]  p.[Gln1201Pro] | Sec39 | - | 4 |
| **NBAS 143** | c.[6859G>T]  p.[Asp2287Tyr] | C-terminal | c.[3860del]  p.[Leu1287*] |  | 6 | 2 |
| **NBAS 147** | c.[451G>A]  p.[Glu151Lys] | ß-propeller | c.[680A>C]  p.[His227Pro] | ß-propeller | 7 | 2 |
| **NBAS 148** | c.[6147-1G>T]  p.[?] | C-terminal | c.[del exon 27-38]  p.[del exon 27-38] |  | 13 | 2 |
| **NBAS 149** | c.[1549C>T]  p.[Arg517Cys] | between ß-Propeller and Sec39 | c.[4843C>T]  p.[Arg1615*] |  | 2 | 1 |
| **NBAS 155** | c.[2617C>T]  p.[Arg873Trp] | Sec39 | c.[5741G>A]  p.[Arg1914His] | C-terminal | - | 5 |
| **NBAS 156** | c.[7003C>T]  p.[Arg2335Trp] | C-terminal | c.[1193A>G]  p.[Asp398Gly] | ß-propeller | 1 | - |

**Supplemental Table S1:** All individuals with NBAS deficiency included in the clinical evaluation of hepatic crises upon treatment including N-acetylcysteine (NAC) compared to treatment without NAC. For each individual PID, genetic variants including affected region or domain and number of episodes each with and without treatment with NAC.

PID patient identification number; NBAS neuroblastoma amplified sequence; NAC N-acetylcysteine; n number; w/o without.

|  |  | | **All individuals with NBAS deficiency (n=16 individuals)** | | | | **Individuals with ILFS2 syndrome (n=7 individuals)** | | | |
| --- | --- | --- | --- | --- | --- | --- | --- | --- | --- | --- |
|  |  |  | **Therapy with NAC**  **(n=49 episodes)** | | **Therapy without NAC**  **(n=37 episodes)** | | **Therapy with NAC (n=10 episodes)** | | **Therapy without NAC**  **(n=25 episodes)** | |
| **Length of hospital stay (days)** | Mean | (SD) | 11.09 | (20.11) | 7.06 | (37.04) | 9.65 | (4.69) | 6.91 | (3.98) |
|  | Median | (Min-Max) | 7 | (0-112) | 6 | (0-21) | 8 | (4-19.5) | 7 | (0-16) |
| **Admission to the ICU** | F/n | (%) | 14/45 | (31.11 %) | 9/32 | (28.13 %) | 4/9 | (44.44 %) | 7/21 | (33.33 %) |
| **Deceased during crisis** | F/n | (%) | 1/49 | (2.04 %) | 1/36 | (2.78 %) | 0/10 | (0 %) | 1/23 | (4.35 %) |
| **ALF** | F/n | (%) | 14/46 | (30.43 %) | 18/35 | (51.43 %) | 5/10 | (50 %) | 13/23 | (56.52 %) |
| **INR** | Mean | (SD) | 2.79 | (3.17) | 3.73 | (4.85) | 3.19 | (3.09) | 4.68 | (5.68) |
|  | Median | (Min-Max) | 1.4 | (1-16) | 1.85 | (1.01-22.3) | 1.45 | (1.14-10) | 2.18 | (1.01-22.3) |
| **AST (U/l)** | Mean | (SD) | 4766.81 | (6150.65) | 5927.4 | (6606.40) | 7194.36 | (6425.50) | 6576.98 | (7150.68) |
|  | Median | (Min-Max) | 1915 | (69-27201) | 3920 | (96-26662.2) | 5190 | (228-21417.6) | 4965.5 | (96-26662.2) |
| **ALT (U/l)** | Mean | (SD) | 3521.77 | (3684.69) | 4208.70 | (4206.63) | 5155.18 | (3876.81) | 4461.24 | (4527.97) |
|  | Median | (Min-Max) | 1937.5 | (56-13521.6) | 3343.8 | (75-19207.2) | 3483.5 | (529.8-13521.6) | 3498.3 | (75-19207.2) |
| **Age at onset (months)** | Mean | (SD) | 41.72 | (21.09) | 36.73 | (37.04) | 61.6 | (19.32) | 30.48 | (12.66) |
|  | Median | (Min-Max) | 41 | (0.07-92) | 27 | (7-192) | 61.5 | (12.66) | 28 | (10-61) |
| **Fever** | F/n | (%) | 44/49 | (89.80 %) | 33/35 | (94.29 %) | 10/10 | (100 %) | 23/23 | (100 %) |
| **Vomiting** | F/n | (%) | 36/48 | (75.00 %) | 27/31 | (87.10 %) | 9/10 | (90 %) | 20/20 | (100 %) |
| **Drowsiness** | F/n | (%) | 21/47 | (44.68 %) | 15/30 | (50.00 %) | 4/9 | (44.44 %) | 10/19 | (52.63 %) |
| **Likely viral/bacterial** | F/n | (%) | 28 viral  13 bacterial | (57.14 %)  (26.53 %) | 20 viral  9 bacterial | (55.55 %)  (25.00 %) | 6 viral  3 bacterial | (60 %)  (30 %) | 15 viral  4 bacterial | (60 %)  (16 %) |

| **Therapy of episodes** | | | | | | | | | | |
| --- | --- | --- | --- | --- | --- | --- | --- | --- | --- | --- |
| **Antipyretics** | F/n | (%) | 38/45 | (84.44 %) | 27/33 | (81.81 %) | 9/9 | (100 %) | 21/21 | (100 %) |
| **Specify** | F/n | (%) | 13 M  19 PCM  13 Ibuprofen | (28.89 %)  (42.22 %)  (28.89 %) | 12 M  13 PCM  12 Ibuprofen | (36.36 %)  (39.39 %)  (36.36 %) |  |  |  |  |
| **Dextrose i.v.** | F/n | (%) | 38/48 | (79.17 %) | 24/26 | (92.31 %) | 9/9 | (100 %) | 17/18 | (94.44 %) |
| **Dextrose i.v. (g/kg/d)** | Mean | (SD) | 9.26 | (2.82) | 9.84 | (2.61) | 9.76 | (1.05) | 10.37 | (1.03) |
|  | Median | (Min-Max) | 10 | (8-11.5) | 10 | (8.6-12) | 10 | (8-11.5) | 10 | (8.6-12) |
| **Lipids i.v.** | F/n | (%) | 23/43 | (53.49 %) | 13/26 | (50.00 %) | 5/8 | (62.5 %) | 10/19 | (52.63 %) |
| **Lipids i.v. (g/kg/d)** | Mean | (SD) | 1.43 | (0.53) | 1.23 | (0.41) | 1 | (0) | 1 | (0.17) |
|  | Median | (Min-Max) | 1 | (1-1) | 1 | (1-1.5) | 1 | (1-1) | 1 | 1-1,5 |
| **Vitamin K** | F/n | (%) | 28/48 | (58.33 %) | 21/30 | (0.00 %) | 5/10 | (50 %) | 13/21 | (61.90 %) |
| **NAC outpatient 150 mg/kg/d** | F/n | (%) | 21/39 | (53.85 %) | / | | 2/8 | (25 %) | / | |
| **NAC oral PCM intoxication regimen** | F/n | (%) | 11/38 | (28.95 %) | / | | 3/8 | (37.5 %) | / | |
| **NAC i.v. PCM intoxication regimen** | F/n | (%) | 30/48 | (62.50 %) | / | | 10/10 | (100 %) | / | |

**Supplemental Table S2:** Hepatic crises in individuals with NBAS deficiency (all individuals and only individuals with ILFS2 syndrome) upon emergency treatment with N-acetylcysteine (NAC) compared to emergency treatment without NAC. Frequencies with percentages or mean values with variance and median with range are given.

NBAS neuroblastoma amplified sequence; ILFS2 infantile liver failure syndrome type 2; n number; NAC N-acetylcysteine; F frequency; SD standard deviation; Min minimum; Max maximum; ICU intensive care unit; ALF acute liver failure; INR international normalized ratio; AST aspartate aminotransferase; ALT alanine aminotransferase; GGT gamma glutamyl transferase; i.v. intravenous; M metamizole; PCM paracetamol.

|  | **Total (n)** | **ALF (frequency (percentage))** | **p-value** | **ALF upon treatment with NAC (frequency (percentage))** | **ALF without treatment with NAC (frequency (percentage))** | **p-value** |
| --- | --- | --- | --- | --- | --- | --- |
| **Suspected bacterial infection** | 18 | 9/18 (50.00 %) | 0.51 | 3/10 (30.00 %) | 6/8 (75.00 %) | 0.058 |
| **Suspected viral infection** | 44 | 18/44 (40.91 %) |  | 8/25 (32.00 %) | 10/19 (52.63 %) | 0.168 |
| **Influenza infection** | 8 | 6/8 (75.00 %) | 0.03 * | 3/4 (75.00 %) | 3/4 (75.00 %) | 1 |
| **Viral infection other than influenza** | 36 | 12/36 (33.33 %) |  | 5/21 (23.91 %) | 7/15 (46.67 %) | 0.15 |
| **SARS-CoV2 infection** | 5 | 2/5 (40.00 %) |  | 0/3 (0.00 %) | 2/2 (100.00 %) | - |
| **Entero-/ Rhinovirus infection** | 3 | 1/3 (33.33 %) |  | 1/3 (33.33 %) | - | - |
| **Rotavirus infection** | 2 | 1/2 (50.00 %) |  | 1/1 (100.00 %) | 0/1 (50.00 %) | - |

**Supplemental Table S3:** Triggering pathogens for hepatic crises in NBAS deficiency including frequency of ALF in dependency of the pathogen and treatment with N-acetylcysteine. For comparing group differences Chi²-tests were performed and p-values are given, p-values <0.05 are considered significant and marked with a star.

ALF acute liver failure, NAC N-acetylcysteine

| **PID** | **Treatment protocol** | **Number of crises** | **Age (months)** | | **Length of hospital stay (days)** | | **ALF** | | **INR** | | **AST (U/l)** | | **ALT (U/l)** | |
| --- | --- | --- | --- | --- | --- | --- | --- | --- | --- | --- | --- | --- | --- | --- |
|  |  |  | **mean** | **SD** | **mean** | **SD** | **frequency** | **percentage** | **mean** | **SD** | **mean** | **SD** | **mean** | **SD** |
| **53** | NAC | 2 | 52.00 | 8.49 | 12.50 | 4.95 | 2/2 | 100 % | 6.00 | 0.91 | 11277 | 3511.49 | 6813 | 1021.13 |
|  | w/o NAC | 3 | 34.33 | 5.51 | 9.67 | 4.16 | 3/3 | 100 % | 6.50 | 7.93 | 5487 | 7708.93 | 3615 | 4836.58 |
| **84** | NAC | 6 | 72.17 | 12.42 | 7.50 | 2.26 | 1/6 | 16.67 % | 2.70 | 3.57 | 6502 | 7907.06 | 5160 | 4962.59 |
|  | w/o NAC | 11 | 35.36 | 15.49 | 5.90 | 3.51 | 2/10 | 20 % | 2.00 | 1.87 | 5446 | 8330.78 | 4376 | 5864.54 |
| **143** | NAC | 6 | 15.67 | 4.03 | 7.67 | 2.58 | 0/6 | 0 % | 1.70 | 0.00 | 2029 | 1323.84 | 2169 | 1016.97 |
|  | w/o NAC | 2 | 10.00 | 4.24 | 6.00 | / | 2/2 | 100 % | 2.20 | 0.20 | 5394 | 557.20 | 4710 | 410.12 |
| **147** | NAC | 7 | 54.14 | 12.95 | 5.28 | 2.43 | 3/7 | 42.86 % | 2.80 | 3.17 | 6165 | 6182.08 | 3414 | 3753.04 |
|  | w/o NAC | 2 | 33.00 | 8.49 | 7.50 | 4.95 | 1/2 | 50 % | 2.20 | 1.48 | 9062 | 12293.76 | 5003 | 3753.04 |
| **148** | NAC | 13 | 38.15 | 10.42 | 1.85 | 2.64 | 0/13 | 0 % | 1.20 | 0.09 | 690 | 584.97 | 747 | 480.90 |
|  | w/o NAC | 2 | 22.00 | 2.83 | 4.00 | 0 | 0/2 | 0 % | 1.30 | 0.11 | 1470 | 202.23 | 1097 | 84.15 |

**Supplemental Table S4:** Comparison of hepatic crises in single individuals with NBAS deficiency treated with and without N-acetylcysteine (NAC). Mean values with standard deviation or frequencies with percentage are given as appropriate.

NBAS neuroblastoma amplified sequence; PID patient identification number; NAC N-acetylcysteine; w/o without; SD standard deviation; ALF acute liver failure; INR international normalized ratio; AST aspartate aminotransferase; ALT alanine aminotransferase.

| **Protein.ID** | **Gene** | **comparison.label** | **logFC** | **pvalue.limma** | **fdr.limma** | **hit** |
| --- | --- | --- | --- | --- | --- | --- |
| P18850 | ATF6 | Control 40°C vs Control 37°C | 0.34 | 0.22 | 0.46 | False |
| P18850 | ATF6 | NBAS 2 40°C vs NBAS 2 37°C | 0.12 | 0.67 | 0.81 | False |
| P18850 | ATF6 | NBAS 5 40°C vs NBAS 5 37°C | 0.41 | 0.14 | 0.28 | False |
| P18850 | ATF6 | NBAS 2 37°C vs Control 37°C | 0.45 | 0.11 | 0.14 | False |
| P18850 | ATF6 | NBAS 5 37°C vs Control 37°C | -0.09 | 0.75 | 0.83 | False |
| P18850 | ATF6 | NBAS 2 40°C vs Control 40°C | 0.23 | 0.39 | 0.46 | False |
| P18850 | ATF6 | NBAS 5 40°C vs Control 40°C | -0.01 | 0.97 | 0.98 | False |

**Supplemental Table S5:** Differential cyclic AMP-dependent transcription factor ATF-6 alpha protein abundance analysis of NBAS-deficient and control fibroblasts from proteomic analysis. Proteins were quantified by TMT-based proteomics and analysed using the limma framework. Log2 fold changes are shown relative to control samples at the corresponding temperature. Adjusted p-values (FDR) were calculated using multiple testing correction. Proteins were considered significant with FDR < 0.05 and absolute fold change > 1.5.


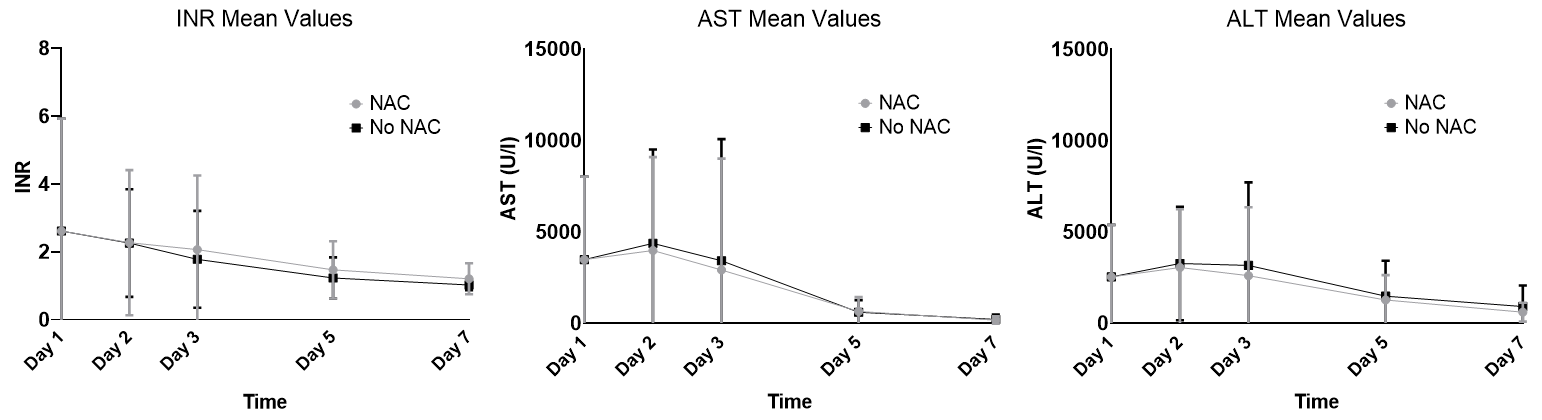


**Supplemental Figure S1:** The development of the laboratory values INR, AST and ALT is shown for crises treated with NAC (at least one day prior treatment before determination of the laboratory value) compared to crises which were not treated with NAC at least one day prior to taking the blood sample.

Mean values and standard deviations are shown as development over time from day 1 to day 7 for all hepatic crises in individuals with NBAS deficiency.

NAC N-acetylcysteine, INR international normalized ratio, AST aspartate aminotransferase, ALT alanine aminotransferase

**Supplemental Figure S2:** Frequency of suspected or proven pathogens in all hepatic crises in NBAS deficiency (a) compared to frequencies of pathogens in only crises fulfilling the ALF criteria (b).

ALF Acute liver failure.


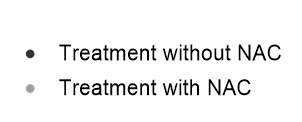


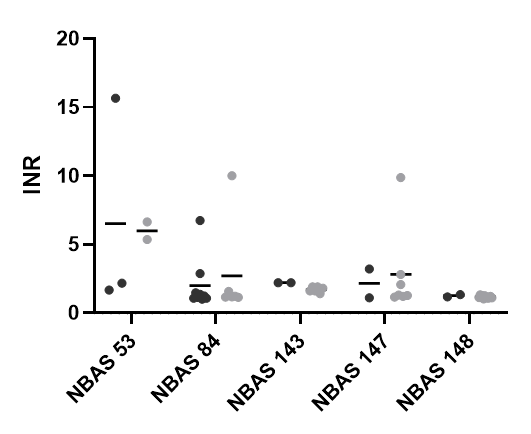


**Supplemental Figure S3:** The laboratory values INR, AST and ALT for all five individuals with NBAS deficiency and at least 2 crises each upon treatment with and without NAC are shown together with the mean values.

NAC N-acetylcysteine, INR international normalized ratio, AST aspartate aminotransferase, ALT alanine aminotransferase


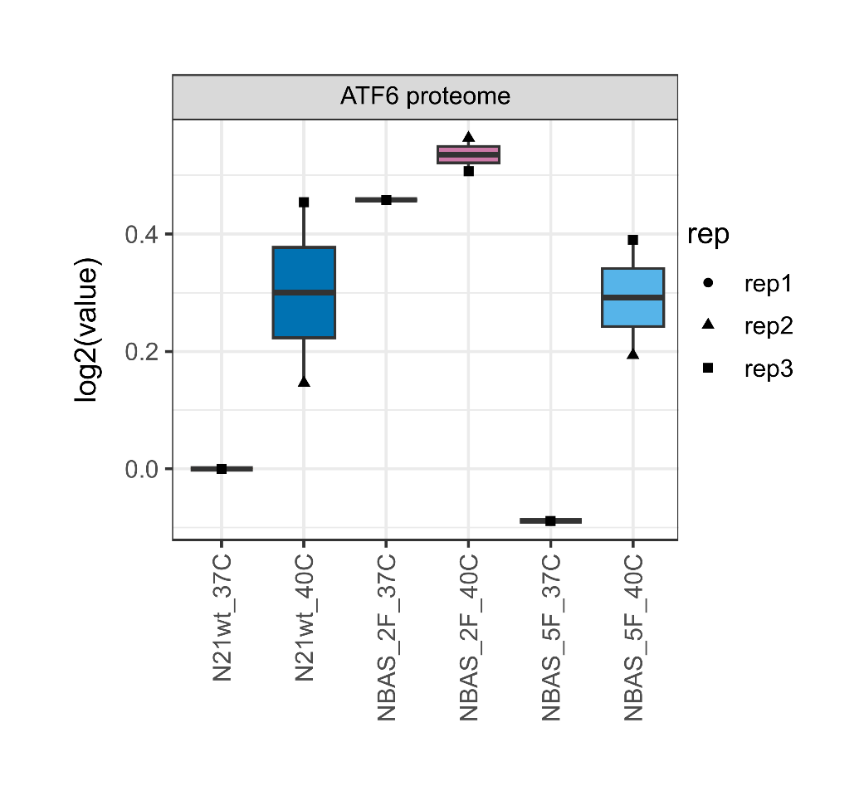


Control 37°C

Control 40°C

NBAS 2 37°C

NBAS 2 40°C

NBAS 5 37°C

NBAS 5 40°C

**Supplemental Figure S4: ATF6 protein levels in NBAS-deficient fibroblasts under basal and fever-mimicking conditions.** Relative abundance of ATF6 was quantified by TMT-based proteomics in control and NBAS-deficient fibroblast cell lines (Sec39: 2F, 5F) cultured at 37°C and 40°C. Protein levels at 37°C were compared to control cells at 37°C, and levels at 40°C were compared to control cells at 40°C. Data are presented as box-and-whisker plots showing three biological replicates per condition. Statistical analysis was performed using the limma framework with multiple testing correction. No significant differences were observed (FDR < 0.05 and absolute fold change > 1.5).
